# Rapid PRRSV-2 ORF5-based lineage classification using Nextclade

**DOI:** 10.3389/fvets.2024.1419340

**Published:** 2024-09-13

**Authors:** Michael A. Zeller, Jennifer Chang, Giovani Trevisan, Rodger G. Main, Phillip C. Gauger, Jianqiang Zhang

**Affiliations:** ^1^Department of Veterinary Diagnostic and Production Animal Medicine, Iowa State University, Ames, IA, United States; ^2^Vaccine and Infectious Disease Division, Fred Hutchinson Cancer Institute, Seattle, WA, United States

**Keywords:** PRRSV, ORF5, classification, lineage, sublineage

## Abstract

Porcine reproductive and respiratory syndrome virus (PRRSV) continues to be a global challenge for swine health. Yim-Im et al. 2023 provides a standard genetic nomenclature, extending previously published works to better characterize PRRSV-2 ORF5-based genetic lineages on a global scale. To facilitate the use of this nomenclature, scaffold sequences, including historical and contemporary vaccines, were synthesized into a dataset designed for Nextclade v3.0. Metadata from the scaffold sequences representing year, country, and RFLP typing of the sequence were incorporated into the dataset. These scaffold sequences were processed through the Augur pipeline using DQ478308.1 as a reference strain for rooting and comparison. The resultant classifier can be accessed through the Nextclade website (https://clades.nextstrain.org/) or a link on the PRRSView homepage (https://prrsv.vdl.iastate.edu/). The resultant classifier functions the same as other classifiers hosted by the Nextclade core group and can provide phylogenetic-based PRRSV-2 ORF5 classifications on demand. Nextclade provides additional sequence metrics such as classification quality and notable mutations relative to the reference. The submitted sequences are grafted to the reference tree using phylogenetic placement, allowing for comparison to nearby sequences of reference viruses and vaccine strains. Additional comparisons between sequences can be made with metadata incorporated in the dataset. Although Nextclade is hosted as a webtool, the sequences are not uploaded to a server, and all analysis stay strictly confidential to the user. This work provides a standardized, trivial workflow facilitated by Nextclade to rapidly assign lineage classifications to PRRSV-2, identify mutations of interest, and compare contemporary strains to relevant vaccines.

## 1 Introduction

Porcine reproductive and respiratory syndrome (PRRS) is the most economically important swine disease in the United States of America, with industry impact being estimated to cost over $600 million annually (1). PRRS is characterized by reproductive failure and late-term abortion in sows, respiratory diseases in all age of pigs, and occasional neurological signs (2–4). The PRRS virus (PRRSV) was first identified as an etiological agent in both the United States and the Netherlands in 1991–1992 (5, 6), although the initial emergence is believed to have occurred earlier. Taxonomically, PRRSV is currently classified into two species: *Betaarterivirus americense* (with virus name PRRSV-2, previously known as North American genotype) and *Betaarterivirus europensis* (with virus name PRRSV-1, previously known as European genotype). In the United States, both species are detected but the majority of detections and economic loss are caused by PRRSV-2 (7).

In 1998, restriction fragment length polymorphism (RFLP) typing based on the cutting patterns of PRRSV-2 ORF5 by three restriction enzymes *MluI*, *HincII*, and *SacII* was introduced to differentiate the RespPRRSV/Ingelvac PRRS MLV vaccine virus (RFLP 2-5-2) from wild-type strains (8). Since then, RFLP typing has been widely used in North America to describe PRRSV-2 genetic diversity (9). However, it has become evident that RFLP itself has limited usefulness in indicating if two strains of PRRSV-2 are genetically similar or distinct (9, 10). In 2010, an ORF5-based phylogenetic classification was proposed to describe PRRSV-2 genetic relatedness and diversity (11). In that study, nine lineages (L1–L9) and 37 sublineages were proposed based on analysis of 8,624 global PRRSV-2 ORF5 sequences. Although the 9-lineage system was adopted for use worldwide, the proposed 37-sublineage system has not been globally used. Lineage 1 was the dominant detection circulating in the United States over the past decade and continues to diversify. To better characterize lineage 1 viruses, in 2019, Paploski et al. (12) divided lineage 1 into L1A–L1E based on analysis of 4,390 U.S. PRRSV-2 ORF5 sequences, with L1D being divided into L1D alpha and L1D beta. In 2021, the same research group added three more sublineages L1F–L1H after analyzing 21,211 U.S. PRRSV-2 ORF5 sequences (13). Yim-Im et al. (14) further refined the phylogenetic classification of PRRSV-2, building upon the prior classification schemes but utilizing a substantially expanded dataset comprising 82,237 global ORF5 sequences. The latest iteration classified global PRRSV-2 ORF5 sequences into 11 lineages (L1–L11) and 21 sublineages (L1A–L1F, L1H–L1J, L5A–L5B, L8A–L8E, and L9A–L9E), with each lineage representing a monophyletic clade (14). Additionally, prior sublineages were revised. For example, L1G was redefined as part of L1B and the use of L1G is discontinued, L1D alpha was redefined as part of L1E with the discontinued use of L1D alpha, and L1D beta was renamed as L1D with the discontinued use of L1D beta (14). Lineages within the latest iteration had within clade distances typically <11% and between clade distances typically >11%. Despite the best effort to be maximally comprehensive, PRRSV variants are continually detected that do not fall into the classification scheme.

To facilitate the ease of use and access to the Yim-Im et al. 2023 nomenclature system, the current report introduces a Nextclade v3 dataset designed from the scaffold sequences. Nextclade is a web-based bioinformatics tool for rapidly assigning lineages to provided unknown sequences (15). Nextclade v3 employs the reconstruction of phylogenetic tree using neighbor-joining methods, whereas the prior version v2 relied on phylogenetic placement methods for a scaffold tree. The benefit of using a Nextclade dataset is that both the method and the reference set become consistent, with the only changing factor being the sequences submitted for classification. The creation of this dataset aims to increase the uptake of a lineage-based nomenclature for PRRSV classification. With this dataset both researchers and swine health care experts will be able to rapidly identify the strain of PRRSV infecting herds and formulate an evidence-based approach in response.

## 2 Method

Scaffold sequences consisting of historical and contemporary PRRSV-2 ORF5 were provided (*n* = 1,100) (14), defining 11 lineages and multiple sub-lineages. Metadata specifying collection date, country, state, and RFLP typing were extracted from the dataset based on the structure of the FASTA def line. Missing information was designated as “unknown.” Six PRRSV-2 modified live virus (MLV) vaccines were additionally included in the dataset: Fostera PRRS, Ingelvac PRRS ATP, Ingelvac PRRS MLV, Prevacent PRRS, Prime Pac PRRS, and PRRSGard.

These scaffold sequences representing PRRSV lineages were processed through the Nextstrain Augur pipeline (16). PRRSV-2 ORF5 DQ478308.1 was used as a reference sequence as the ORF5 is genetically identical to the PRRSV-2 prototype VR-2332 strain. The VR-2332 is historical and serves as a root to all lineages defined by Yim-Im et al. 2023. The sequences were aligned with MAFFT under automatic settings using the Augur align function. A tree was built via IQ-TREE (17) under a general time-reversible model (18), rooted to DQ478308.1 using the corresponding Augur tree and refine functions. The initial tree was refined, and nucleotide and amino acid mutation information was inferred via the Augur ancestral and translate functions, and additional metadata was added to the tree as traits. Metadata and tree were combined and exported from Augur into a Nextclade JSON tree. When users submit data for classification, query sequences are first compared against the reference sequence DQ478308.1 to identify mutations, which are then compared with branch-defining mutations of every node and tip in the reference phylogenetic tree. This process facilitates a fast initial placement of the query sequences onto the reference tree. As of Nextclade v3, an additional analytical step has been introduced to re-evaluate the relationships among the query sequences with each other. This step aims to resolve local phylogenetic relationships more accurately, thereby further refining the overall phylogenetic tree. The output scaffold tree was hosted on a public GitHub repository for use as an external Nextclade dataset (see Data Availability). A link has been added onto the ISU *PRRSView* web resource^1^ to facilitate access.

## 3 Results

**Figure 1:**
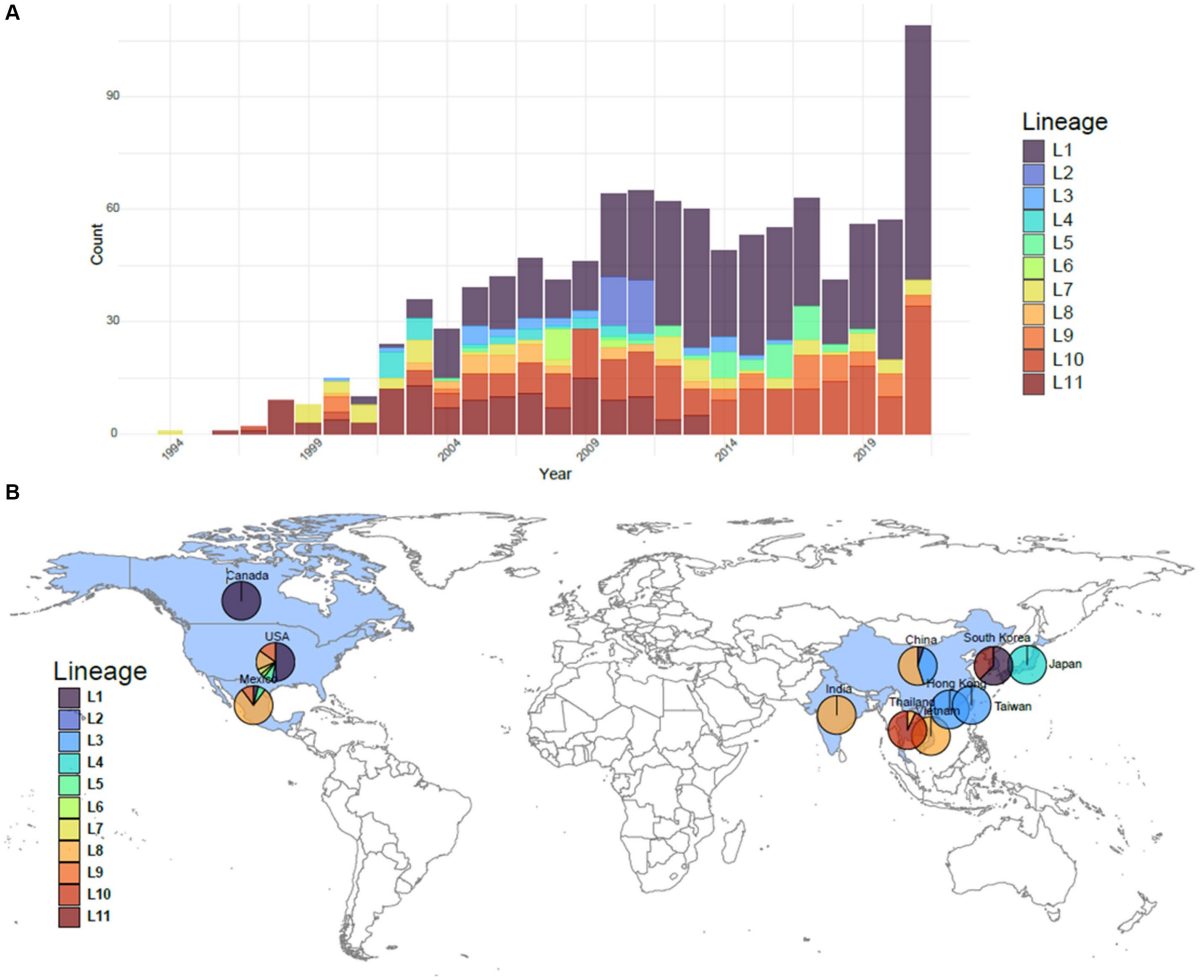
Overview of the PRRSV-2 ORF5 lineages comprising the 1,100 sequences in the reference dataset. **(A)** The number per year of each lineage represented in the dataset. **(B)** The geographic breakdown of where sequences originated. Sequences originated from 10 countries colored in blue: Canada, China, India, Japan, Mexico, South Korea, Taiwan, Thailand, the United States, and Vietnam. Breakdowns of lineages from these countries used in each dataset are shown as a pie chart.

The reference dataset was comprised of 1,100 PRRSV-2 ORF5 sequences from 1994 to 2021, collected from 10 different countries: Canada, China, India, Japan, Mexico, South Korea, Taiwan, Thailand, the United States, and Vietnam (Figure 1) (14). The majority of the sequences originate from the United States (77%), with a large contingency representing Asia (17%). Lineage 1 has broad genetic diversity and multiple sublineages in circulation, and is represented in 466 sequences (42%). Lineage 8 and lineage 9 are also well represented in this dataset with 219 and 133 sequences, respectively (20 and 12%, respectively). All other lineages have less than 10% representation but greater than 2% representation. The smallest lineage, lineage 11, has 24 representative sequences.

**Figure 2:**
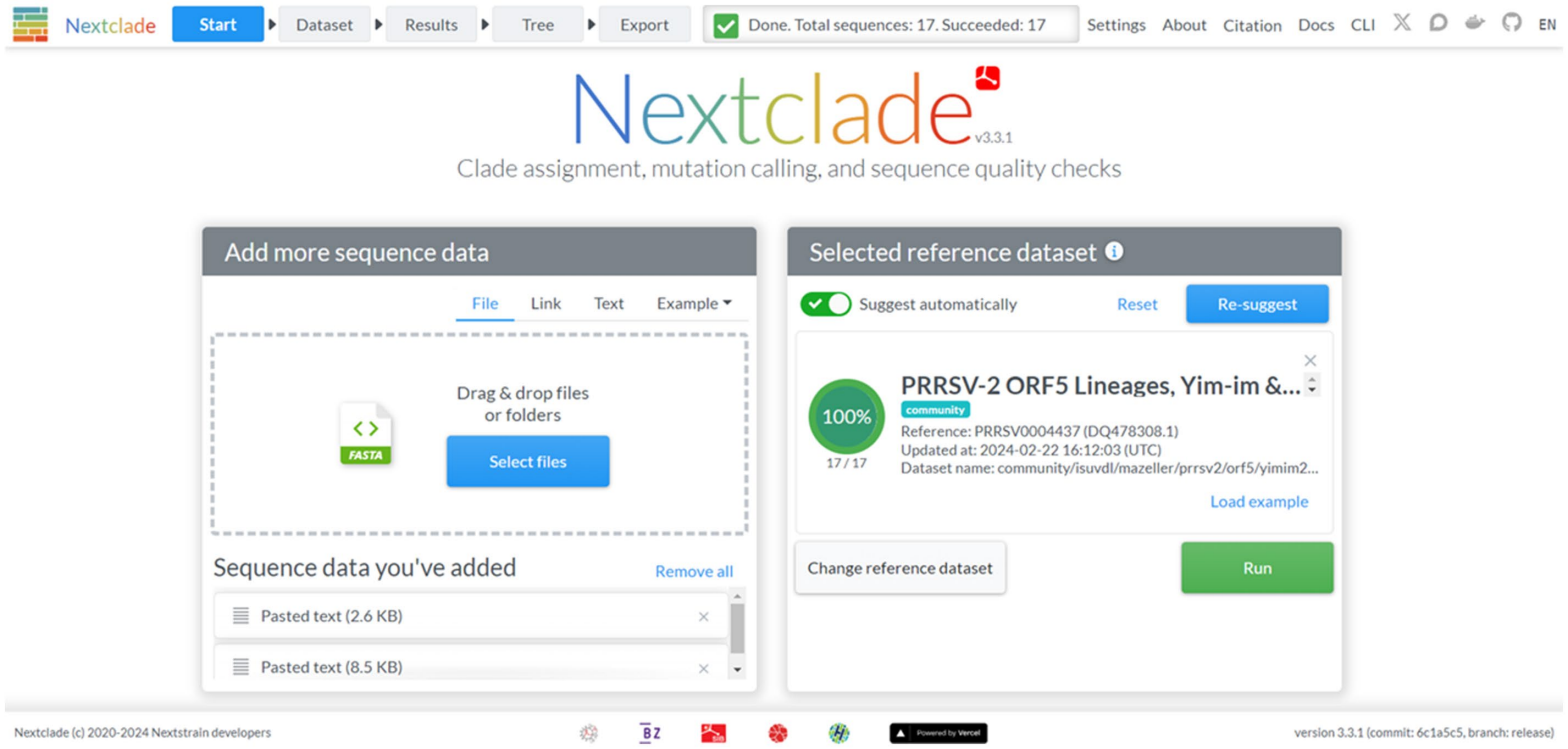
The start screen of the Nextclade web platform, indicated by the navigation bar at the top. The reference dataset has been set to PRRSV-2 ORF5 lineages.

**Figure 3:**
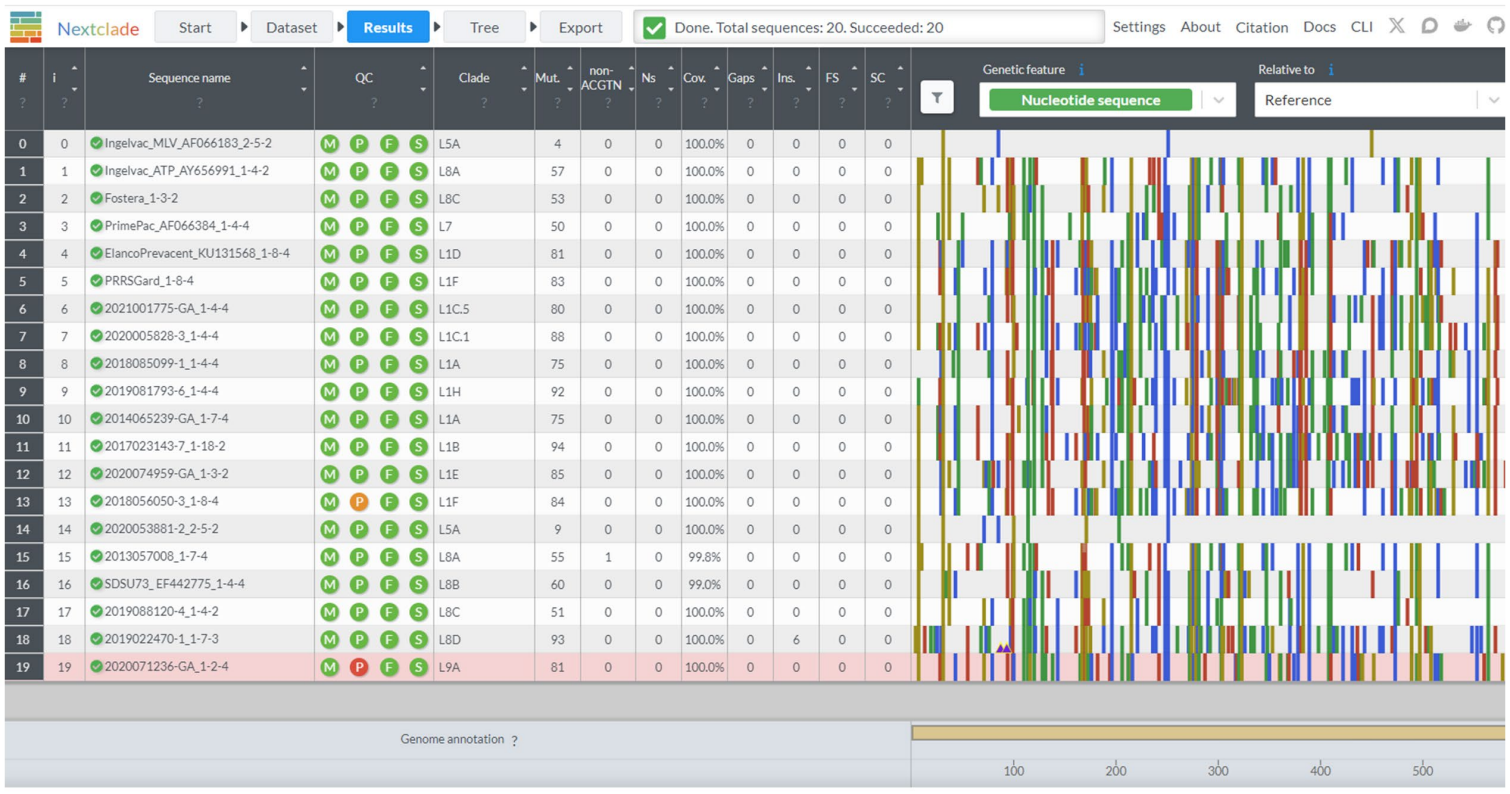
The results screen of the Nextclade web platform, indicated by the navigation bar at the top. Sequence name, lineage assignment, and sequence quality metrics are displayed in tabular format. Additional beneficial information is displayed such as sequence duplication in the sequence name column, and single nucleotide polymorphisms from the base genome on the graph to the chart to the right.

**Figure 4:**
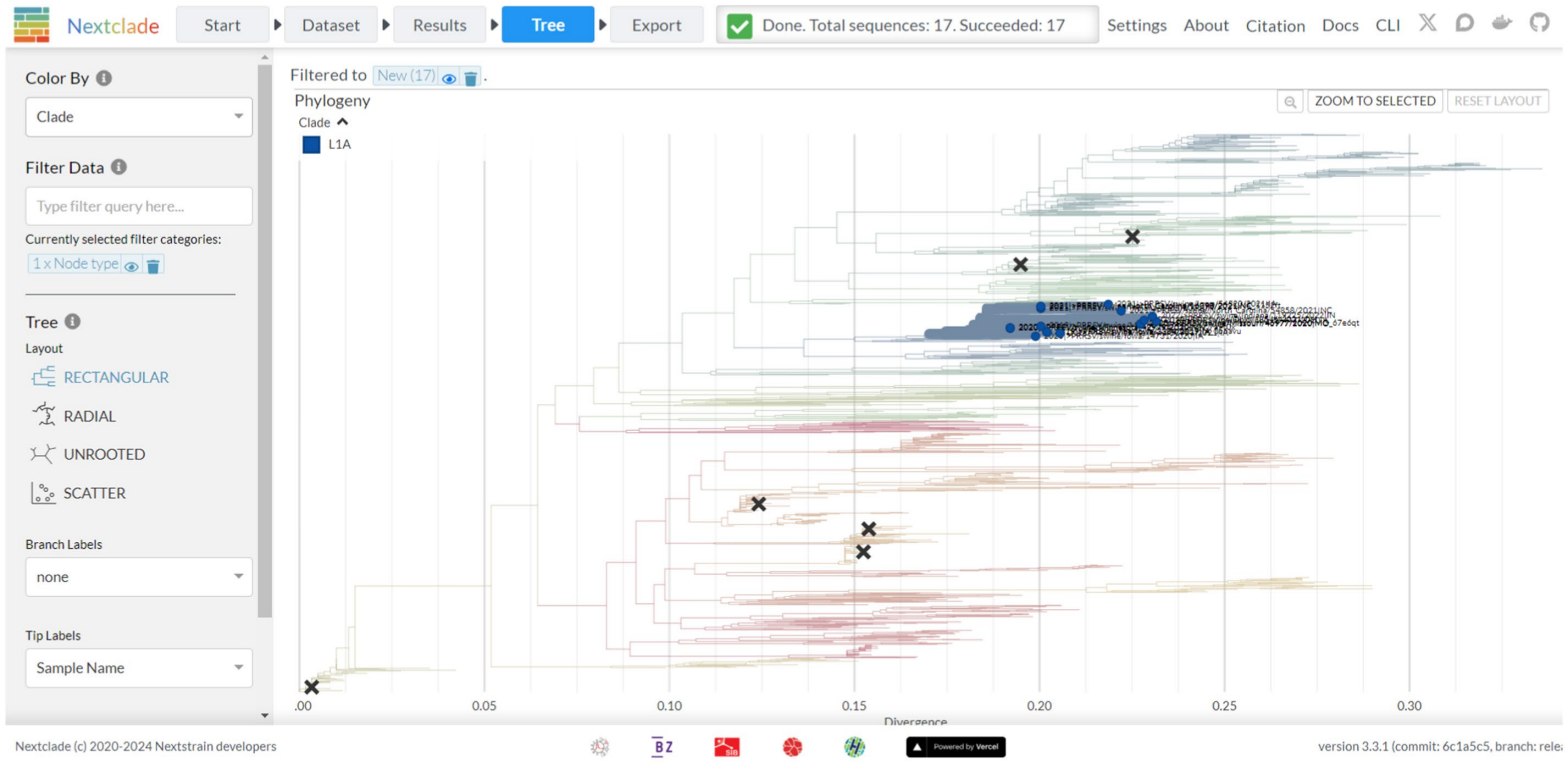
The tree screen of the Nextclade web platform, as indicated by the navigation bar at the top. This screen displays a feature rich phylogenetic tree where branches are colored based on phylogenetic lineage, new strains of interest are emphasized, and vaccine strains are denoted through X’s marked on the tree. Additional display and filter options are available from the panel on the left-hand side of the tree.

Nextclad provides a drag-and-drop interface for users to locally upload FASTA containing PRRSV-2 ORF5 for classification (Figure 2). After sequence submission, Nextclade provides the user with a tabulated view of the submitted sequences and assigned clades via tree inference (Figure 3). The assigned clade column refers to ORF5 lineage in this instance. This screen provides additional information for the quality control of the sequence classification, flagging sequences that do not meet a minimal quality control criterion. Information about coverage of the sequence, the number of unknowns, presence of stop codons, and frameshifts are displayed in the table (Figure 3). Additionally, mutations in the ORF5 relative to the reference in terms of both nucleotide and amino acid can be displayed on the right and sequence view column. All data summarized in the tabular view can be downloaded as a CSV, as well as the alignment and a phylogenetic tree with the input sequences placed on it (Figure 4).

A tree view is available via the tree tab, presenting a tree with features similar to other trees viewed via Auspice (Figure 4). By default, the tree is colored by lineage as defined by Yim-Im et al. 2023. The tree is rooted to DQ478308 and marked with six black X’s denoting six MLV vaccine strains. By default, the tree is filtered based on new sequences but can be filtered for any metadata information provided prior, including nucleotide and amino-acid mutations. The tree can be recolored to denote the metadata for year, country, state, RFLP, and nucleotide or amino acid genotypes per sequence. Sequences can additionally be colored by their quality control metric. The tree can be viewed as either rectangular, radial, or unrooted. Amino acid mutations between the strains can be visualized as branch labels.

Nextclad provides a diversity chart below the tree that displays diversity as either a raw count of mutation events or entropy per sequence position as either amino acid or nucleotide. This chart indicates that codon position 58 has the highest diversity, which when visualized does not correspond to a specific lineage. The diversity chart indicates relatively higher and consistent diversity at the 5′ end up to codon 61, where after the diversity is on average lower.

## 4 Discussion

Nextclade provides a standardized, facilitated workflow to rapidly assign lineage classifications to PRRSV-2 ORF5 containing sequences. Both the method of classification and the reference set are packaged together, producing a highly consistent protocol. The only change between runs by different users are the input sequences for classification, leaving the scaffold tree the same and consistent. An advantage is that Nextclade eliminates the need to disseminate FASTA references between parties, and the method becomes standardized. The richness of features provided by Nextclade are suitable for both swine producers and researchers for classification and comparison of their PRRSV-2 ORF5 sequences. Particularly, Nextclade allows end users to rapidly compare their strains to contemporary vaccines and allows scientists to identify mutations of interest for strains in circulation.

There are a couple of limitations in the use of this dataset for PRRSV classification. While Yim-Im et al. 2023 have gone through considerable effort to create the most globally inclusive dataset to date, there will continue to be sequences that fail to classify, representing unsampled diversity missed in global surveillance. Additionally, as an RNA virus, PRRSV is rapidly evolving with a mean rate between 4.7 × 10^−2^ and 1.55 × 10^−3^ substitutions/site/year (13, 14). PRRSV ORF5 sequences will drift from the confines of the current classifications, and it will be necessary to update the PRRSV Nextclade dataset in the future to remain a relevant source for sequence classification. The public nature of the Yim-Im et al. 2023 nomenclature and this Nextclade dataset provides a foundation for members within the swine health community to readily extend the PRRSV-2 ORF5 nomenclature as is needed.

The ability to include and visualize metadata in Nextclade is especially important. The inclusion of vaccine strains on the tree allows users to rapidly identify if a well-matched vaccine option exists relative to their strain. Additionally, producers can upload distinct FASTA files containing sequences from different farms, production flows or region and get a consistent tree structure that allows a consistent structured format for between and within farm, production flow or region comparisons. In the case of farm-derived vaccines, both the vaccine strain and the strains of concern can be visualized together on the same tree. The ability to color PRRSV by country of origin shows that some PRRSV has diversified based on geographic region, with the U.S. hosting the most diversity globally. As such, observing the tree by country can potentially make it easier to notice cross-country spillovers. Perhaps the most interesting bit of metadata is the inclusion of RFLP. The mixing of RFLPs in the dataset are indicative of how little descriptive power RFLPs provide at the genetic level. The most common RFLPs observed, such as 1-4-4, have the largest genetic diversity and fail to describe a monophyletic genetic lineage.

While PRRSV-2 ORF5 has historically been used to classify lineages (8, 11), this gene only represents approximately 4% of the entire viral genome. Other researchers have developed additional nomenclature systems such as for the whole genome (19), but lineages for other genes of interest are not established at present. As future interest grows in whole genome sequencing and characterizing genes beyond ORF5, Nextclade can provide the ability to classify these other genomic regions given well curated datasets are made available with reference sequences for those specific genome regions.

PRRS remains a major swine disease for the foreseeable future. Rapid techniques for classifying different lineages of PRRSV-2 are imperative for a rational response to the disease, whether through the implementation of biosecurity measures or the induction of immunity via commercial MLV vaccines, farm-tailored live virus inoculum, or farm-specific autogenous vaccines. Nextclade provides a distinct utility by wrapping both the method and reference set into a single package, which is a powerful benefit that can be extended and applied to other major viral diagnostic diseases.

## Data Availability

This web resource is accessible from Nextclade directly (https://clades.nextstrain.org/) or from ISU PRRSView (http://prrsv.vdl.iastate.edu). The datasets generated for this study can be directly accessed in the Nextclade GitHub repository (https://github.com/nextstrain/nextclade_data/tree/master/data/community/isuvdl/mazeller/prrsv2/orf5/yimim2023).

## Footnotes

^1^https://prrsv.vdl.iastate.edu/

## References

[1] Holtkamp D. Rotto H. Garcia R.. (2007). The economic cost of major health challenges in large U.S. swine production systems. American Association of Swine Veterinarians. Orlando, FL. 85–89.

[2] Zimmerman JJ Dee SA Holtkamp DJ Murtaugh MP Stadejek T Stevenson GW. Porcine reproductive and respiratory syndrome viruses (porcine arteriviruses) Diseases of swine. (2019). 685–708.

[3] Cao J Li B Fang L Chen H Xiao S. Pathogenesis of nonsuppurative encephalitis caused by highly pathogenic porcine reproductive and respiratory syndrome virus. J Vet Diagn Invest. (2012) 24:767–71. doi: 10.1177/1040638712445764, PMID: 22585954

[4] Rawal G Almeida MN Gauger PC Zimmerman JJ Ye F Rademacher CJ. *In vivo* and *in vitro* characterization of the recently emergent PRRSV 1-4-4 L1C variant (L1C.5) in comparison with other PRRSV-2 lineage 1 isolates. Viruses. (2023) 15:2233. doi: 10.3390/v15112233, PMID: 38005910 PMC10674456

[5] Wensvoort G Terpstra C Pol JM ter Laak EA Bloemraad M de Kluyver EP. Mystery swine disease in The Netherlands: the isolation of Lelystad virus. Vet Q. (1991) 13:121–30. doi: 10.1080/01652176.1991.9694296 1835211

[6] Collins JE Benfield DA Christianson WT Harris L Hennings JC Shaw DP. Isolation of swine infertility and respiratory syndrome virus (isolate ATCC VR-2332) in North America and experimental reproduction of the disease in gnotobiotic pigs. J Vet Diagn Invest. (1992) 4:117–26. doi: 10.1177/104063879200400201, PMID: 1616975

[7] Trevisan G Linhares LCM Crim B Dubey P Schwartz KJ Burrough ER. Macroepidemiological aspects of porcine reproductive and respiratory syndrome virus detection by major United States veterinary diagnostic laboratories over time, age group, and specimen. PLoS One. (2019) 14:e0223544. doi: 10.1371/journal.pone.0223544, PMID: 31618236 PMC6795434

[8] Wesley RD Mengeling WL Lager KM Clouser DF Landgraf JG Frey ML. Differentiation of a porcine reproductive and respiratory syndrome virus vaccine strain from North American field strains by restriction fragment length polymorphism analysis of ORF 5. J Vet Diagn Invest. (1998) 10:140–4. doi: 10.1177/104063879801000204, PMID: 9576340

[9] Trevisan G Sharma A Gauger P Harmon KM Zhang J Main R. PRRSV2 genetic diversity defined by RFLP patterns in the United States from 2007 to 2019. J Vet Diagn Invest. (2021) 33:920–31. doi: 10.1177/10406387211027221, PMID: 34180734 PMC8366264

[10] Cha SH Chang CC Yoon KJ. Instability of the restriction fragment length polymorphism pattern of open reading frame 5 of porcine reproductive and respiratory syndrome virus during sequential pig-to-pig passages. J Clin Microbiol. (2004) 42:4462–7. doi: 10.1128/JCM.42.10.4462-4467.2004, PMID: 15472294 PMC522335

[11] Shi M Lam TTY Hon CC Murtaugh MP Davies PR Hui RKH. Phylogeny-based evolutionary, demographical, and geographical dissection of North American type 2 porcine reproductive and respiratory syndrome viruses. J Virol. (2010) 84:8700–11. doi: 10.1128/JVI.02551-09, PMID: 20554771 PMC2919017

[12] Paploski IAD Corzo C Rovira A Murtaugh MP Sanhueza JM Vilalta C. Temporal dynamics of co-circulating lineages of porcine reproductive and respiratory syndrome virus. Front Microbiol. (2019) 10:2486. doi: 10.3389/fmicb.2019.02486, PMID: 31736919 PMC6839445

[13] Paploski IA Pamornchainavakul N Makau DN Rovira A Corzo CA Schroeder DC. Phylogenetic structure and sequential dominance of sub-lineages of PRRSV type-2 lineage 1 in the United States. Vaccine. (2021) 9:608. doi: 10.3390/vaccines9060608, PMID: 34198904 PMC8229766

[14] Yim-Im W Anderson TK IAD P VanderWaal K Gauger P Krueger K. Refining PRRSV-2 genetic classification based on global ORF5 sequences and investigation of their geographic distributions and temporal changes. Microbiol Spectr. (2023) 11:e0291623. doi: 10.1128/spectrum.02916-23, PMID: 37933982 PMC10848785

[15] Aksamentov I Roemer C Hodcroft E Neher R. Nextclade: clade assignment, mutation calling and quality control for viral genomes. J Open Source Softw. (2021) 6:3773. doi: 10.21105/joss.03773

[16] Huddleston J Hadfield J Sibley T Lee J Fay K Ilcisin M. Augur: a bioinformatics toolkit for phylogenetic analyses of human pathogens. J Open Source Softw. (2021) 6:2906. doi: 10.21105/joss.02906, PMID: 34189396 PMC8237802

[17] Nguyen L-T Schmidt HA von Haeseler A Minh BQ. IQ-TREE: a fast and effective stochastic algorithm for estimating maximum-likelihood phylogenies. Mol Biol Evol. (2015) 32:268–74. doi: 10.1093/molbev/msu300, PMID: 25371430 PMC4271533

[18] Tavaré S. Some probabilistic and statistical problems in the analysis of DNA sequences In: Lectures on mathematics in the life sciences. Rhode Island: American Mathematical Society. (1986). 57–86.

[19] Guo J Liu Z Tong X Wang Z Xu S Chen Q. Evolutionary dynamics of type 2 porcine reproductive and respiratory syndrome virus by whole-genome analysis. Viruses. (2021) 13:2469. doi: 10.3390/v13122469, PMID: 34960738 PMC8706008

